# Sailing Into Uncharted Territory: Victim Survivors’ Perspectives on Some of the Tensions of Engagement as Partners in Co-Design Research

**DOI:** 10.1177/10778012251362227

**Published:** 2025-07-30

**Authors:** Katie Lamb, Renee Fiolet, Kelly Broadhurst, Heshani S. De Silva, Kelsey Hegarty

**Affiliations:** 1 2281 The University of Melbourne, Parkville, Melbourne, VIC, Australia; 2 Centre for Quality and Patient Safety Research, Institute for Health Transformation, 2104 Deakin University, Melbourne, VIC, Australia; 3 Member of WEAVERS Lived Experience Group, 2281 The University of Melbourne, Parkville, Melbourne, VIC, Australia; 4 The Royal Women's Hospital, Melbourne, VIC, Australia

**Keywords:** lived experience, domestic violence, qualitative research, co-design, victim survivors

## Abstract

The engagement of people with lived experience as co-researchers is gaining momentum across areas of physical and mental health and community services research. Reporting of co-designed research often espouses the benefits and successes, without critical reflections on how the process was experienced by people with lived experience. This paper explores the experiences of victim survivor co-researchers on a research team at an Australian university, with a focus on tensions. Four themes were identified: Boundaries: clarity about how we use our space; Representation: the weight of our voice; Reform: the pace of change; and Recognition: converting lived expertise into academic currency.

## Introduction

The field of knowledge production has experienced a shift in recognition of the importance of ensuring research is informed by and relevant to real world contexts (Vaughn & Jacquez, 2020). This has seen a challenge to historically extractive research practices (Daryani et al., 2022) and greater interest in research developed in partnership with the groups being studied (Peters et al., 2024; Vaughn & Jacquez, 2020). The approach of integrating researcher theoretical and methodological expertise with real world knowledge and experiences has been described as a “mutually reinforcing partnership” (Cargo & Mercer, 2008, p. 327).

This movement has increasingly led to the use of research approaches which are described as participatory, coproduced, or co-designed. Participatory or coproduced research is being undertaken in a range of sectors including physical health (McGeon et al., 2023; Oliver et al., 2019) and mental health (King & Gillard, 2019), as well as with Indigenous populations internationally (Baum & Simpson, 2006; Jull et al., 2018). The take up is strongly underpinned by a view that people who have been impacted by the issues being explored often have a unique understanding as a result of their lived experience (Lonbay et al., 2021). This is a concept that aligns well with feminist frameworks which underpin much research and service responses to domestic, family, and sexual violence (Johnson & Flynn, 2021). The term “expert by experience” is often used to capture this expertise (Fox, 2022; Lamb et al., 2020; Law & Morrison, 2014).

Standardized language or definitions of “co-designed” or “co-produced” research are elusive (Masterson et al., 2022; McGill et al., 2022; Thomas-Hughes, 2018) and also make systematic reviews in this space challenging (Slattery et al., 2020). However, there is general agreement in the literature that that these approaches challenge traditional power dynamics by recognizing and valuing the expertise of lived experience as a source of expertise (Darby, 2017; McGeon et al., 2023). There is also general consensus that engagement of people with lived experience in research occurs across a continuum (Slattery et al., 2020). These continuums vary but generally range from low levels of engagement, where people with lived experience have limited engagement or degree of influence over the project, to work that aspires to greater involvement, where work is initiated and led by people with lived experience (Lamb et al., 2023; Werner-Seidler & Shaw, 2019).

While some researchers are exploring ways to more formally and systematically embed people with lived experience in the research process, the practice is still far from widespread. Documentation of the initiatives that have been undertaken often engage in “celebratory reporting” when outlining coproduction efforts undertaken rather than offering any “reflexive accounting” of how that process went and any challenges faced (Howard & Thomas-Hughes, 2021). Reviews of co-design in nonresearch settings have found that traditional timelines are often not adequate for co-design projects and that co-researchers can experience frustration waiting to see their input lead to change (Tindall et al., 2021).

While some insights can be gained from reflections of those who have been involved in co-design research with lived experience co-researchers in others areas such as health (King & Gillard, 2019; Papageorgiou et al., 2023), there is less information available in the domestic, family, and sexual violence field. While some papers have outlined the benefits of engaging victim survivors as co-researchers (Cullen et al., 2023) and the principles for good practice (Perot et al., 2018), the literature suggests that less has been written to offer insights into co-researcher experiences, processes behind the work, necessary conditions or infrastructure, or the inherent tensions of engagement in such processes (Oliver et al., 2019). Building on our previous paper (Fiolet et al., 2024), which outlined the motivations and benefits for victim survivors who become involved in our lived experience co-design group, this paper focuses on the key tensions people with lived experience identified. The research question guiding this paper is:

What are victim survivors’ perspectives on the key tensions that arise when engaging as co-researchers in the context of domestic, family, and sexual violence?

### About the WEAVERS Group

The Safer Families Centre of Research Excellence (the Safer Families Centre) at the University of Melbourne, Australia, established a lived experience co-design group called the WEAVERS in 2016. This initiative was designed to ensure the voices of women who have experienced domestic, family, and sexual violence shape research, teaching and training at the University using co-design methods. At the time that the WEAVERS group was established, it was rare for victim survivors to have formal input into both setting the research agenda and co-designing, implementing, and disseminating findings of research about domestic, family, and sexual violence.

The group initially comprised nine women who had been participants in previous research studies concerning abuse and violence run by the Safer Families Centre. Recruitment for the group now occurs periodically when spaces become available. Membership of the group was 20 at the time this study was undertaken, but additional more recent recruitment has raised the membership to 30 women. These women are located across Australia and are aged 25–67. Recruitment for the group was conducted via social media advertising over a number of platforms. A detailed interview and self-reflection and induction process then occurred before engagement in the group. The group was supported by an administrative support person (a victim survivor who is a member of the group) and an academic researcher who coordinated the group, as well as a lived experience researcher who provided peer support sessions. All work completed by members of the group was paid at the university research assistant rate. While rates were consistent, payment methods differed between group members according to individual circumstances. Work was completed in person and online and the women self-selected into projects they were interested or had relevant lived experience in.

Women in the WEAVERS group came from a range of backgrounds. Our group defined lived experience as someone who has experienced “any form of domestic, family, intimate partner or sexual violence” as a child or an adult and brings their “expertise by experience” to their work. The group was engaged in research projects funded through competitive grants but also engaged in research commissioned by nongovernment and government organizations.

Members of the lived experience co-design group were called “co-researchers,” as they worked alongside academic researchers in the broader Safer Families Centre, primarily on projects that use co-design approaches. Members of the group were also involved in research priority setting, attending planning days, and contributing to decisions about which research projects the team should seek funding for. The term “co-researchers” is used to reflect the team's way of working where researchers and people with lived experience work in partnership, rather than victim survivors sitting outside the team in an advisory role. Co-design was conceptualized by the group as research that was jointly designed and carried out by victim survivor co-researchers and academic researchers. The group had co-designed two key documents designed to record the group's practice and method of working but also to promote discussions about best practice across the broader domestic, family, and sexual violence research sector (Lamb et al., 2020, 2023). The WEAVERS group has supported the development of many research projects at other universities across Australia and regularly delivers webinars and presentations at local and international conferences and for the World Health Organisation.

## Method

Following ethics approval from The University of Melbourne Human Research Ethics Committee (Reference Number 2023-25756-36238-3), all 20 victim survivors who were members of the WEAVERS group were asked to participate in semistructured qualitative interviews about their experiences of being involved (via Zoom or phone). Interview questions asked about what their experience of being in the group was like; what their motivations were for joining the group were; what they found beneficial about being part of the group; and any challenges they experienced while in the group. They were also asked for any suggestions they might have for improvements to the way the group runs to support future planning. Interviews were conducted by Zoom and phone by an experienced qualitative researcher who had not worked with the group before. The academic researchers were not aware of which members of the group had participated in the interviews. Interviews were generally between 30 and 60 min and held at a time selected by the interviewee.

Interviews were recorded and transcribed then coded using QSR NVivo. Data analysis followed guidelines provided by Braun and Clarke regarding reflexive thematic analysis. This involved the research team acknowledging their own diverse views and values, yet all identifying as feminist researchers (Braun & Clarke, 2006). Data were anonymized by the interviewer and then coded, interpreted, and grouped into themes to create current understandings. Groupings and themes were discussed and refined by both academic researchers and co-researchers. The findings were then written up by a team of academic researchers and members of the WEAVERS group.

## Results

A total of 15 women (of the 20 members at the time) gave informed consent and participated. They were aged between 26 and 74 years and all lived within Australia. They had all been members of the WEAVERS for between three and nine years. They were from a range of cultural backgrounds. No further detail about members is appropriate to provide given the small sample size and potential for public identification.

When victim survivors in the WEAVERS group reflected on their experiences of working in partnership with university researchers, a range of benefits and challenges were described. Some of the challenges described were logistical but others related to inherent tensions of working in this complex way. This paper outlines four themes which summarize the tensions identified: Boundaries: clarity about how we use our space; Representation: the weight of our voice; Reform: the pace of change; and Recognition: converting lived expertise into academic currency.

### Boundaries: Clarity About How We Use Our Space

Group members generally understood that participation in the group was not directly designed to offer peer support but yearning for some form of sharing of experiences was also mentioned by some as a key tension. Members of the group emphasized the importance of establishing boundaries and being clear about the group's purpose and what was expected. They described experiences where they had been in other groups with victim survivors where it had been necessary to have repeated discussions about boundaries and mindfulness about what level of detail you share and the impact this might have on triggering or overwhelming others in the group.

Several WEAVER group members suggested that this reassurance of not having to share your own story had made them feel more comfortable joining the group:

I think probably we all went there with this understanding that we were going to share our stories. We didn’t actually ever do that. I think that may have felt uncomfortable for some women, but I found that really comforting. That I didn’t have to tell my story to be validated as a person who had survived all sorts of different generational and interpersonal violence. (Interview #8)

There were members of the group who did express a desire to know more about the history and experiences of women in the group. One described the lack of space dedicated to hearing each other's personal experiences as “something missing” (Interview #7) and another talked about hoping joining the group would be a way of engaging in a peer support process:

I would pretty much enjoy if the women that are part of that group to have more of a chance to connect a little bit more and have a peer support group happening. But it's not about a peer support group, I understand that. (Interview #13)

However, one group member suggested that she could see benefit in the establishment of more formal processes for peer support around participation in the WEAVERS research activities.

### Representation: The Weight of Our Voice

Another key tension that several participants mentioned was the issue of representation and ensuring that multiple and varied voices are heard within the group. The group comprises a diverse range of women with varied backgrounds, levels of education, lived experience of disability, mental ill health, homelessness, and cultural diversity and members of the LGBTIQ community. Interviewees noted that from its inception, the group was *never* designed to be “representative” or to speak on behalf of any specific marginalized group,

it was challenging for a lot of people to set up the terms of reference because it is not the most exciting part of the process. But it's such an important part of the process because we talked a lot about representation. We were very conscious that we were not a representative group and that we could never be. (Interview #1)

However, the tension of feeling a responsibility to advocate for the needs of their community weighed heavily on some:

Sometimes, I feel like I’m not enough of a voice for [my marginalised community] I guess or enough of a childhood experience voice. I feel like there needs to be more diversity as well, even within that. So it does feel a little bit like…I guess it feels a little bit heavy at times. (Interview #10)

I feel like because of my privilege, I’ve got the responsibility to elevate those voices that still aren’t heard, and it would be really, really great for the WEAVERS to concentrate on those voices and really advocate for the people that are the most vulnerable and aren’t receiving the support that they should be receiving. (Interview #11)

Over the years the group has often come back to this issue and discussed whether the key to hearing more diverse voices is to broaden the group and specifically recruit for diversity or alternatively to set up specific groups for those voices less often heard in domestic, family, and sexual violence research:

I've long term advocated that they set up an Aboriginal WEAVERS and that they set up a gender-diverse and transgender, non-binary WEAVERS, or they include more people from those groups into our groups if they can't set up their own group. (Interview #12)

The issue of ensuring cultural and emotional safety is often considered by the WEAVERS group. Discussions have centered on acknowledging that bringing someone into the group because they are part of a marginalized group can also be problematic if they do not have that expert cultural expertise and support built around them:

but then of course, you know, if you're from a marginalised group, then again, there should never be only one of you on a group because… that puts too much pressure on that one person. (Interview #1)

### Reform: The Pace of Change

The slow movement from research to advocacy to reform changes was a tension for some group members. A number of WEAVERS suggested that when they joined the group they had big dreams of making large scale systemic reform that ultimately were not necessarily achieved by the group:

I expected at the beginning that we were going to change the face of healthcare in Australia, now I realise that we have contributed to improvements in healthcare in Australia. (Interview #1)

The pace of change and structural barriers was described as a frustration for some:

It's hard too, because I think we’ve got such a systemic barrier too here, where there are these ethical procedures and all of this sort of stuff that we need to do in order to produce any form of research that that's what makes and takes the process such a long time. I think just allowing that freedom of speech a little bit more and not being so bureaucratical. (Interview #11)

One described their involvement as co-researchers as a form of activism:

because the amount of work that goes into it, it's almost a qualified form of activism, something that people can't deny. People can walk past a bunch of protestors and just go, ugh. But this can inform policy, and it's powerful. It's powerful activism too. (Interview #15)

Another saw the inclusion of lived experience in research as influencing positive change:

I feel like that is a form of activism because it's empowering activists and getting people with lived experience to be able to make those changes. It is a form of activism by giving a pathway and way forward for people and organisations to use that. (Interview #2)

However, others felt that the model of research funding was too reactive and described wanting to be more active in setting the research and reform agenda:

I feel like some of the work – and this isn’t just with the WEAVERS, but I feel like a lot of the work across the board in family violence as a whole is very responsive. It would be nice for WEAVERS to be a little bit more directive. So, like, we choose what we work on next rather than waiting for something to respond to. (Interview #11)

For some, they believed that research could only be seen as activism or advocacy if it leads to change:

There's doing the work and there's seeing the results of the work and I am very keen to make sure that the work is not done in vain. It's not done in vain but done to achieve the ends it was intended to, which is often systems change right?…How do we make sure that gets the right people to achieve that end. I love that bit. (Interview #14)

### Recognition: Converting Lived Expertise into Academic Currency

A final tension that was described specifically related to the way in which lived experience expertise is recognized and embedded within academic and research institutions. The issue of capacity building and the development of research skills was a complex one as two WEAVERS already had research qualifications, the remaining 18 members of the group did not.

Some of the WEAVERS described feeling like the relationships between the academic researchers and the victim survivor- co-researchers in the team was one of peers:

to just try and start off with the understanding that no one's voice was more or less important or valid, whether they were an academic or not. That was a principle. That was a guiding, founding principle of the group. (Interview #1)

Some members of the group were adamant that they did not feel that they needed academic qualifications alongside their “expertise by experience”:

I don’t have scholastic qualifications [in this]… I don’t have a uni degree. But as I said at one of the conferences. I said, you can only get this from living it and if this was a university course, I assure you no one would do it. (Interview #9)

Others described wanting access to courses and qualifications in order to feel like more equal partners:

I don’t have a research background…I don’t have a degree. So, yeah, some training opportunities or something that helps because I think coming from the prospective of having been in family violence, you get told – well, I got told all the time how dumb I was, how I didn’t know everything. (Interview #6)

Another tension that emerged was around the degree of participation with some WEAVERS wanting piecemeal work that was flexible and could fit around their other work and family commitments:

It's really important that I can do WEAVERS stuff and work for a living as well. (Interview #4)

However, other victim survivors wanted to see more developed career pathways for lived experience co-researchers beyond entry-level research assistant roles:

Some people, this is now their passion, this is now what they want to get out there and want to do to use their experience and it's difficult to do it when you’re trying to pay the bills. It is ad hoc, it is a couple of hours here and there. I think that could be a bit of a barrier to what we could achieve…I can see so much more potential. (Interview #2)

Suggestions included the development of research training pathways broader than just PhDs for people with lived experience, as well as setting up similar roles at all universities doing research into domestic, family, and sexual violence and resourced in a way that allows for a viable career option rather than something that needs to be balanced with other work:

I’m a single mum…I would like to do a PhD, but I don’t have, you know, I’m not sitting on a pile of cash with a ton of time to do a PhD. (Interview #14)

I think it would be incredible to have courses that the WEAVERS could do so that then they, if they were like me – I didn’t have any qualifications – so that they would then gain a qualification in the area they want to work I’ve always wanted to study and further my education so I think that would be an amazing thing. (Interview #9)

## Discussion

The movement towards greater democratization of research and knowledge production has been advocated by researchers (Barke et al., 2022; Edwards & Brannelly, 2017). Approaches that value lived expertise fit particularly well with a feminist approach which underpins much of the development of services and research in the domestic, family and sexual violence sectors (Hague & Mullender, 2006; Johnson & Flynn, 2021). Feminist researchers often focus on exploration of ways to ensure victim-survivors are centered as legitimate sources of knowledge in research about domestic, family and sexual violence (Campbell & Wasco, 2000; Davies, 2021). This paper reports on some of the tensions described in interviews conducted with 15 victim survivors of domestic, family and sexual violence who have been working as co-researchers with a team of academic researchers at the University of Melbourne. Four themes were identified which related to *boundaries, representation, reform, and recognition*. Below we contextualize these findings and describe some ongoing responses and actions to these tensions by the group.

A tension that the WEAVERS identified was of *boundaries* with varied perspectives on how they see the group's purpose, even when it had been the focus of discussion. For some, participation is focused on the work, dissemination, and advocacy, while others are also looking for the group to provide support (both formally and informally). Previous involvement in survivor advocacy had led some members of the group to want firm boundaries around the degree of disclosure and personal sharing of experience of violence and abuse as an element of supporting their wellbeing and willingness to participate. The importance of establishing parameters around sharing of traumatic information has been noted by others engaged in co-production with people who have experienced trauma (McGeon et al., 2023). To address this issue in the WEAVERS group, a set of self-reflection questions has been prepared for all new members of the group to consider before making a decision about whether to join. The questions ask survivors to consider whether they have enough resources in place both personally and professionally to prevent their health and wellbeing being negatively impacted; consideration of boundaries they have in place; and their own assessment of their readiness to participate (See Box 1).

Box 1. Self-Reflection Questions (People with Lived Experience) (Lamb et al. 2023).

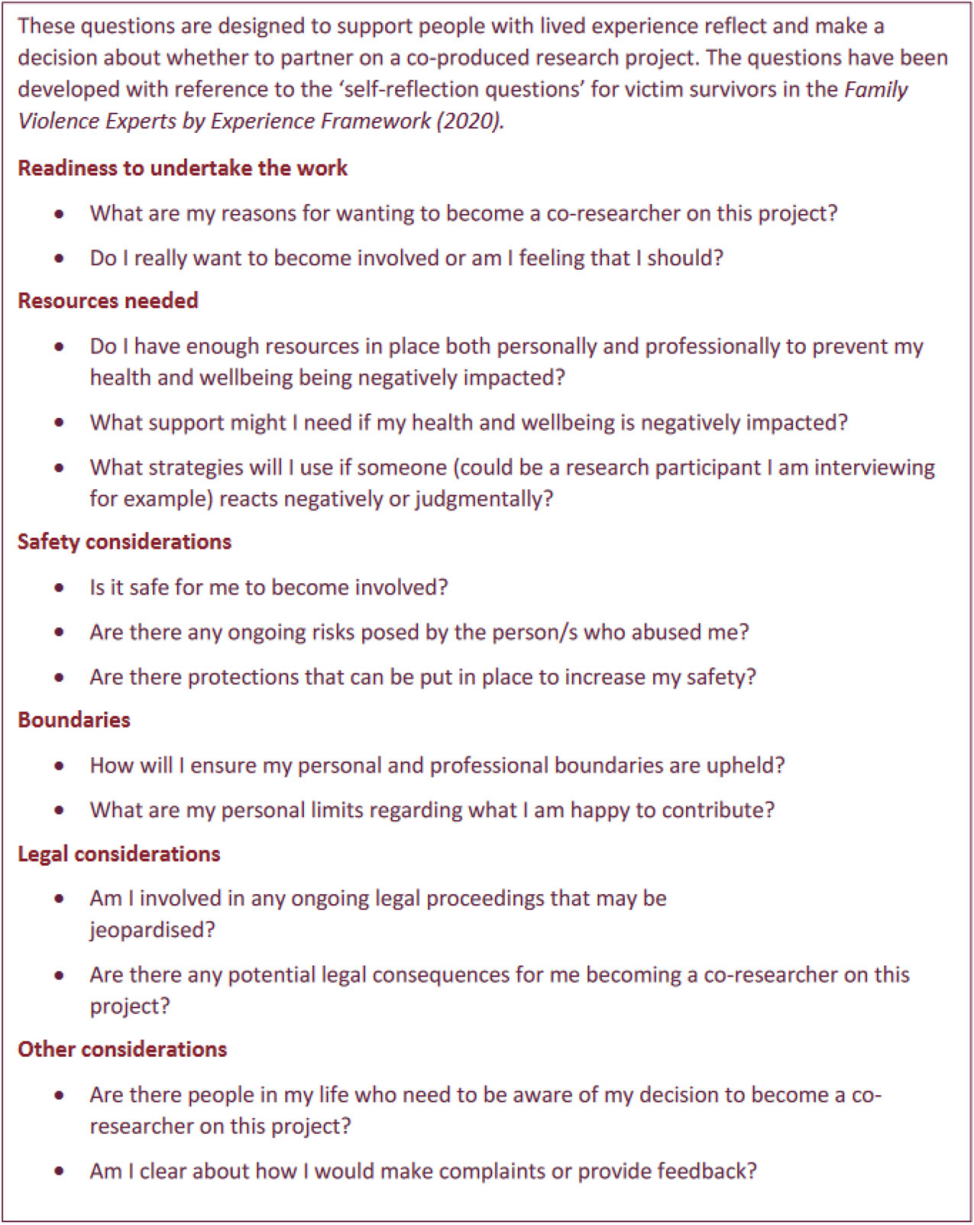

It is important to note that in recognition of the importance of preparing those working *with* people with lived experience (Wheildon et al., 2023), a similar set of questions has also been developed for researchers to assess their readiness to engage (with acknowledgement that there will be researchers who also have lived experience and the two categories are not mutually exclusive) (See Box 2). A member of the group has also drafted a trauma informed aspirations and expectations agreement as part of her work with survivors in other sectors and this is an approach that the WEAVERS group is interested in adopting also.

Box 2. Self-Reflection Questions (Academic/Lived Experience Researchers) (Lamb et al., 2023).

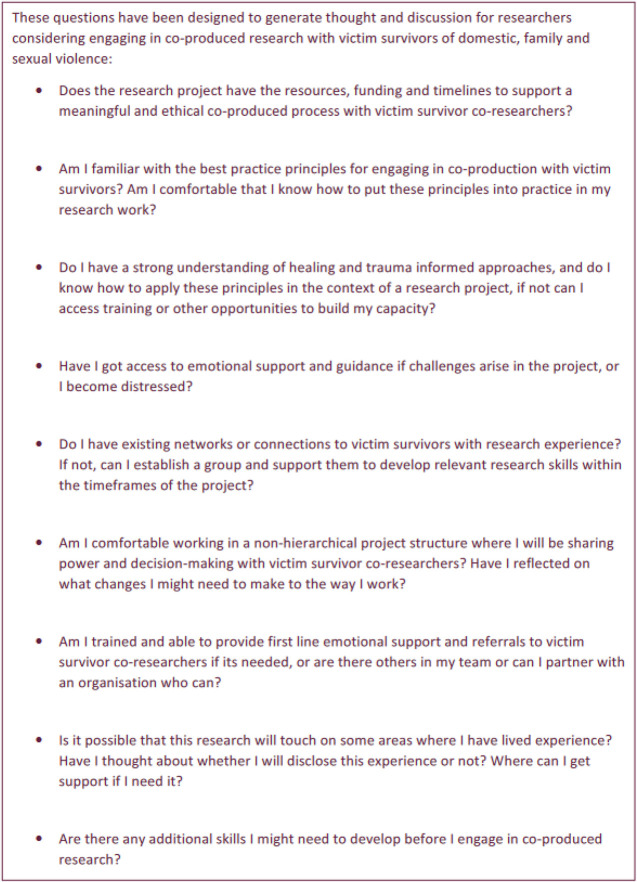

The importance of *representation* was another tension. Ensuring that a diverse range of victim survivor voices and experiences are captured, understood, and amplified is one commonly raised in the literature (Kennedy et al., 2022; Robinson et al., 2023; Zark et al., 2023). Another is the weight that people with lived experience from marginalized communities may feel to represent the views of their entire community even though this is clearly not possible (Dembele et al., 2024). These issues also concerned the WEAVERS. Whether victim survivors from diverse populations should be more actively targeted and recruited or whether parallel groups for diverse victim survivors should be established has been debated within the group. A key principle that has guided decision-making around these issues has been ensuring that victim survivors from marginalized populations are well supported. At times, the group had decided not to actively recruit survivors from certain diverse population groups, even though it was agreed that their perspective would be important because the group was concerned that it did not have adequate cultural competence or expertise to ensure safe participation. This has led to discussions about the potential benefits of partnering with other specialized organizations and is something the group is planning to explore.

An additional tension that emerged in the group was the varied appetite and patience for *reform*. The link between research and reform in the domestic violence area has been noted by previous authors (Haviland et al., 2008). In the current study, some co-researchers saw research as making significant contributions to advocacy, while others wanted to see a stronger link between research, advocacy and change. In some ways while this tension exists, it often plays out in a constructive way. More patient members of the group are sometimes encouraged by more radical actors to ask for and expect more change from key institutions and the group meeting somewhere in the middle. However at other times, it leads to some frustration and lack of satisfaction for those wanting to see faster and more impactful systemic change. As Williams et al. (2020) note, disagreements and difficult conversations might actually be a welcome sign that different views and experiences are being discussed as part of a relational process.

Another key challenge is *recognition* of the shape and form that lived experience research roles should take. In this research, findings suggest that there are two clear categories of co-researchers—those who want to undertake lived experience research work alongside other work and responsibilities and those who want to forge a career as a researcher. Both approaches can cause tension. For those wanting occasional or casual work opportunities, the challenge is for the research coordinator ensuring adequate timely coverage of project work, meetings and in-person events. For the co-researchers it can be difficult to take time away from other paid employment. For those wanting a pathway into a research career, academic requirements for research roles are often rigid and require study at the postgraduate or doctoral level which can be a significant barrier. The challenges of engaging in co-produced research within the confines of academic frameworks and funding regimes has been noted (Barke et al., 2022). The lack of qualifications and pathways specifically available for victim survivors to have their ‘expertise by experience’ recognized, can be a significant roadblock and barrier to further reduction of power imbalances. This is an issue in many areas of lived experience work (Byrne et al., 2017; Fava et al., 2020) and co-produced research but of even greater significance when people with lived experience are from marginalized groups (such as sole parents) and have caregiving responsibilities such as many victim survivors.

It has been suggested that researchers involved in co-designed research with victim survivors of violence and abuse should accommodate a broad range of opportunities for involvement and ensure survivors can shape and evolve their roles (Kennedy et al., 2022). In the mental health sector, discussions are further progressed on ways to support people with lived experience become peer workers and build research careers as co-researchers. As a result, our team has partnered with the ALIVE National Centre for Mental Health Research Translation to share ideas and these include: researchers offering mentoring; running workshops focused on research skill development and supporting academic researchers who have lived experience to break down the silos between people with lived experience and academic researchers (Banfield et al., 2021).

Since the completion of this study, the WEAVERS operating model has been modified to ensure that the needs of both survivors who would like casual opportunities for engagement as well as those who would like to forge a career in research can be supported. This new model is being developed in partnership with a newly formed Steering group of WEAVER members. Unlike the mental health sector where consumer and carer researchers are expected to be on research teams, research about violence and abuse does not yet routinely engage survivors as co-researchers. It has been suggested that progress could be better achieved and measured if reporting on survivor involvement in research became a standard practice required by funding organizations, ethics committees, and external reviewers (Kennedy et al., 2022).

## Conclusion

The team's engagement of victim survivors as co-researchers about domestic, family, and sexual violence has demonstrated considerable benefits for the quality of research produced and also victim survivors (Fiolet et al., 2024). However, the work has at times provided challenges for both victim survivor co-researchers and academic researchers. Our findings identified some of the key tensions that have been experienced by the victim survivors engaged as co-researchers as part of ongoing monitoring of the program. Given the lack of specific evidence and resources available about best practice for embedding victim survivors in research teams to draw on to shape this work, this paper aims to contribute to knowledge and understanding about the complexities while also providing support to others considering engaging in work of this type. Future work could involve interviews with academic researchers to explore their perspectives on some of these challenges and others that arise in their work with victim survivor co-researchers. Additional knowledge about how to safely support diverse and marginalized people with lived experience engage as co-researchers is also needed.
